# 

**Published:** 1887-04

**Authors:** 


CONTENTS:

ARTICLE I.—The General Structure of the Teeth.
By Dr. Carl Heitzmann ........ 529
" II.—Are Micro-Organisms Necessary to Pus Formation.
By G. V. Black, M. D., D. D. S ........ 543
" III.—Courtesy Among Dentists at Professional Meetings.
By J. N. Farrar, M. D., D. D. S ........ 550
" IV.—Transplantation of Teeth. By Wm. N. Morrison, D. D. S. 559
" V.—Treatment of Blind Abscesses. By Dr. Louis Ottofy ........ 562
" VI.—New Method of Mounting an Artificial Denture.
By H. W. Howe, D. D. S ........ 564
" VII.—Runyan's Method of Bridge-Work.
By H. W. Runyan, D. D S ........ 565
" VIII.—Evolution in Pathology ........ 566
The Illinois State Dental Society ........ 569
Missouri State Dental Society ........ 570

EDITORIAL, ETC.

A Singular and Interesting Case ........ 570

History of Dentistry ........ 571

Chicago College of Dental Surgery ........ 572

BIBLIOGRAPHICAL.

The American System of Dentistry ........ 573

MONTHLY SUMMARY.

Resorcin Inoculations in Phlegmon ........ 575

Sedative Cough Mixture ........ 575

Warts ........ 575

Grind It Off ........ 576

